# Radiomics in Stroke Neuroimaging: Techniques, Applications, and Challenges

**DOI:** 10.14336/AD.2020.0421

**Published:** 2021-02-01

**Authors:** Qian Chen, Tianyi Xia, Mingyue Zhang, Nengzhi Xia, Jinjin Liu, Yunjun Yang

**Affiliations:** Department of Radiology, The First Affiliated Hospital of Wenzhou Medical University, Zhejiang, China.

**Keywords:** decision-making, neuroimaging, radiomics, stroke, texture analysis

## Abstract

Stroke is a leading cause of disability and mortality worldwide, resulting in substantial economic costs for post-stroke care each year. Neuroimaging, such as cranial computed tomography or magnetic resonance imaging, is the backbone of stroke management strategies, which can guide treatment decision-making (thrombolysis or hemostasis) at an early stage. With advances in computational technologies, particularly in machine learning, visual image information can now be converted into numerous quantitative features in an objective, repeatable, and high-throughput manner, in a process known as radiomics. Radiomics is mainly used in the field of oncology, which remains an area of active research. Over the past few years, investigators have attempted to apply radiomics to stroke in the hope of gaining benefits similar to those obtained in cancer management, i.e., in promoting the development of personalized precision medicine. Currently, radiomic analysis has shown promise for a variety of applications in stroke, including the diagnosis of stroke lesions, early prediction of outcomes, and evaluation for long-term prognosis. In this article, we elaborate the contributions of radiomics to stroke, as well as the subprocesses and techniques involved in radiomics studies. We also discuss the potential challenges facing its widespread implementation in routine practice and the directions for future research.

Stroke is a vascular event characterized by a sudden onset of focal neurologic deficits in the relevant part of the central nervous system, including ischemic stroke, intracerebral hemorrhage (ICH), and subarachnoid hemorrhage (SAH) [1]. Globally, it represents a leading cause of mortality and disability, and there were an estimated 80 million stroke survivors in 2016, resulting in substantial economic costs for post-stroke care [2, 3]. Despite the declining age-standardized mortality rates over the past 2 decades, the absolute number of incident stroke, disability-adjusted life-years lost due to stroke, and stroke-related deaths is increasing [3]. Along with population growth and aging, the burden of stroke is likely to increase further.

Medical imaging, such as X-ray computed tomography (CT) or magnetic resonance imaging (MRI), promotes the development of personalized diagnosis and treatment in cancer, by complementing with genomic, proteomic, and metabolomic technologies [4, 5]. Neuroimaging as a part of imaging protocols, plays a vital role in stroke analytics in both clinical practice and trials. In acute ischemic stroke (AIS), the results from recent clinical trials with imaging features as criteria for selection of subjects for therapy confirmed the improved efficacy of endovascular thrombectomy compared to standard medical care with intravenous alteplase [6, 7]. Additionally, the perfusion-diffusion mismatch determined by advanced neuroimaging has shown good potential for delayed interventions, wherein thrombolytic treatment for target-penumbra can be undertaken beyond the 4.5 h time point [8]. In acute ICH, some randomized clinical trials (e.g., ATACH, INTERACT, or SPOT-AUST) have been performed, with the goal of improving prognosis for patients with a high risk of early hematoma growth using baseline neuroimaging markers [9-12].

During the past decade, advances in computational technologies, particularly in machine learning, have placed medical imaging in an increasingly central role in patient-specific management. This progress makes it possible to convert subjective visual interpretation into an objective assessment that is driven by image data. Radiomics has emerged in this context. It is a computer-aided process, in which a profuse number of quantitative features (e.g., shape, intensity or texture) can be extracted from biomedical images in an objective, reproducible, and high-throughput manner [13-15]. This process is motivated by the concept that digitally encrypted images contain biologic information related to the pathophysiology of certain diseases, and this information can be exploited via quantitative image analyses [16]. In the oncology field, the potential of radiomics arises from its ability to allow quantitative assessment of intratumor heterogeneity that reflects phenotype and/or microenvironment, which may not be visually perceived [17, 18]; radiomic data, in combination with other medical information such as demographic, clinical, histologic or genomic data, can be used for clinical-decision support systems to improve treatment decision-making and accelerate advancements toward precision medicine in cancer [5, 16, 19, 20].

Radiomics has also shown promise for a variety of applications in stroke, facilitating its personalized management at an early stage. In this review, we describe the workflow of radiomics as well as its basic techniques. We also outline the main contributions of radiomics to stroke, with an emphasis on the diagnostic, predictive, and prognostic value of radiomics during post-stroke care. Finally, we discuss the potential challenges facing the widespread implementation of radiomics in routine clinical practice and the directions for future research.

## 1. Radiomic workflow and techniques

**Figure 1.**
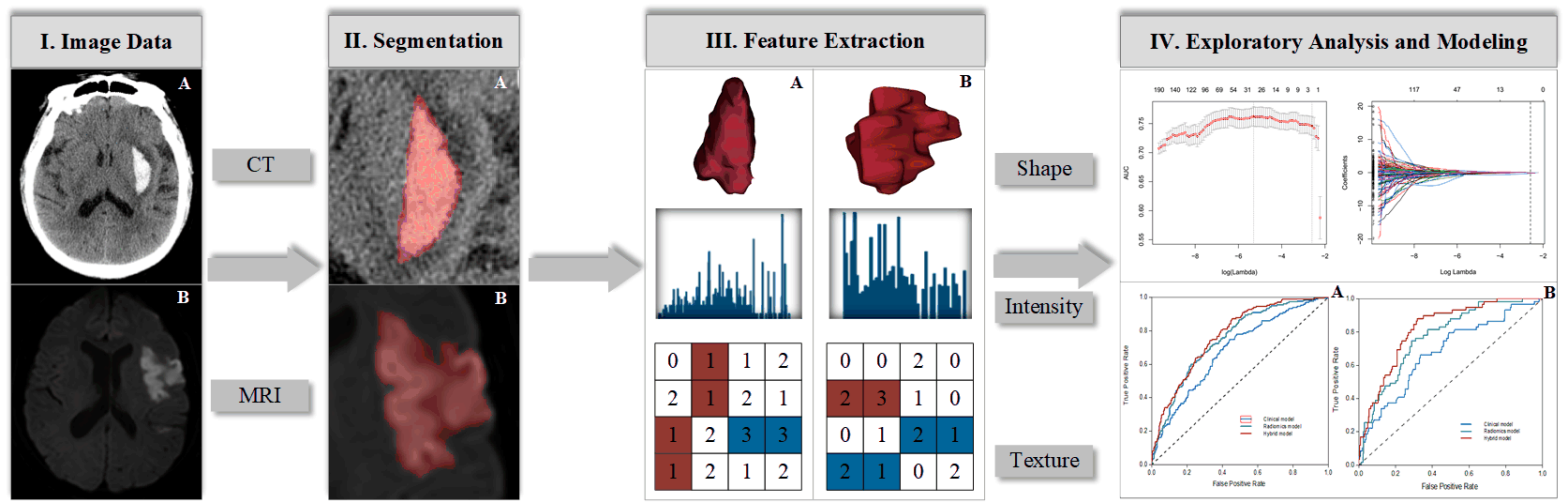
Flowchart shows the typical process of radiomics in stroke neuroimaging. (A) Example CT image of an intracerebral hemorrhage and (B) example MR image of a patient with ischemic stroke.

Radiomics is a high-throughput process, during which a quantitative relationship can be established between multimode data sources by converting medical images into numerous radiomic features. This quantitative relationship is expected to address a clinically relevant problem in certain diseases, such as early diagnosis or accurate prognosis prediction, thereby improving treatment decisions. Regardless of the lesion type and clinical purpose, the workflow of radiomics is similar. Radiomic analysis involves a spectrum of continuous subprocesses, including image data acquisition, segmentation, feature extraction, exploratory analysis, and modeling (Fig. 1).

### 1.1 Image data acquisition

The process of radiomics begins with digital imaging. Radiomic analyses can be applied to all imaging modalities (i.e., CT, MRI, PET, and ultrasound), but CT is the most commonly used technique. This is particularly useful in stroke, as non-contrast CT (NCCT) can rapidly determine the presence of an intracranial hemorrhage [21]. Sufficient imaging data sources are beneficial to statistical inference, but heterogeneity in imaging protocols may cause unexpected effects on both the quality of the extracted features and the radiomic models [22, 23]. For CT images, several studies have recently shown that the majorities of radiomic features are highly affected by image acquisition and reconstruction parameters and thus may be nonreproducible [24-26]. Results from a phantom study have shown that diverse scanners made by different manufacturers could cause variability in radiomic feature values [27]. Additionally, slice thicknesses as well as bin width of gray-level discretization also cause such variation [28].

Efforts are ongoing to enhance the stability of radiomic features via preprocessing techniques, such as normalization of the gray-level and voxel size or resampling of the pixel size to minimize feature dependency on voxel size [29, 30]. Nevertheless, progress has been limited to certain diseases or imaging modalities of the disease. To date, there is still a lack of evidence regarding the stability and reproducibility of radiomic features obtained from CT in either ischemic or hemorrhagic stroke. It should be noted that the robustness of radiomic features and comparability between radiomics studies can only be achieved through extensive disclosure of standardized imaging protocols [20].

### 1.2 Segmentation

Segmentation is the act that isolates a lesion of interest from the surrounding normal tissue. This represents the most critical step during radiomic workflow because it determines which region within an image is to be analyzed further and from where the radiomic feature is generated. This step involves variations in the selection of regions of interest (ROIs) and segmentation techniques.

Generally, the segmentation of ROIs is performed in 3 dimensions (3D)—that is, the entire lesion is extracted and analyzed, although 2D analysis at a single slice can also be carried out [31]. It is unknown how much lesion information should be harnessed to conclude robust results in radiomic analysis. Intuitively, 3D-radiomics could provide more information, particularly for lesions that are spatially heterogeneous and large in volume. However, volumetric assessment is time- and labor-intensive, and more importantly, increases the risk of radiomic feature instability due to segmentation of ROIs from multiple image slices [32]. Although 3D-radiomics appears to be more valuable for evaluating tumor heterogeneity in patients with colorectal cancer [33], in those with other disorders, including stroke, there is little evidence to indicate that 3D-ROI outperforms 2D-ROI. Considering the pros and cons of both schemes, more attention should be paid to efficiency during the process of segmentation.

**Figure 2.**
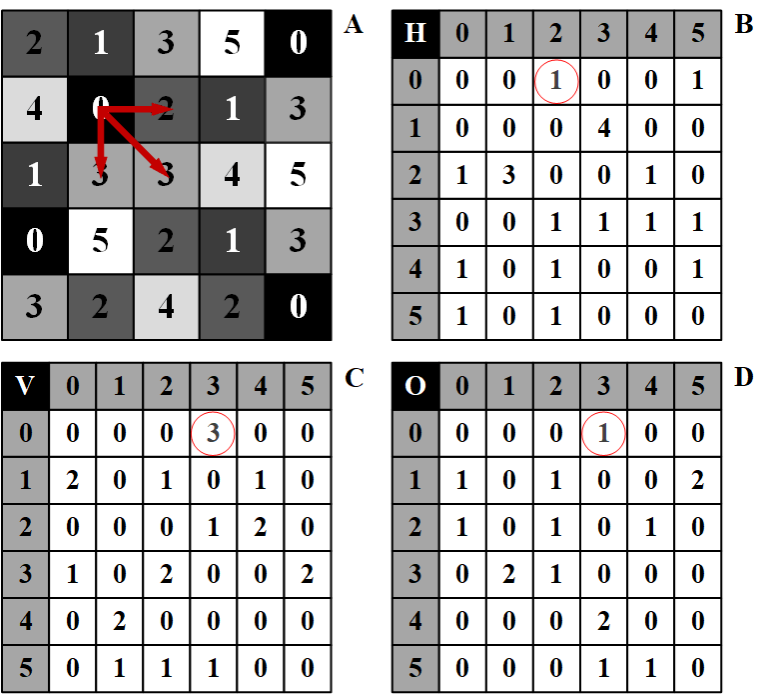
Texture in radiomics. (A) A stylized gray-level image (5 x 5 pixels) with grey values ranging from 0 (black) to 5 (white) and its derived gray-level co-occurrence matrix (GLCM) in horizontal (B), vertical (C), and oblique (D) directions. Row and column numbers in the GLCM represent corresponding gray values, while cells in white contain the number of times corresponding gray values occurs adjacent to each other in three directions. For example, the frequency of gray values of 0, 2, and 3 (red arrows on the gray-level image) is then mapped onto corresponding cell of GLCM (red circles) each time they occur adjacent to each other in particular direction. In texture analysis, GLCM represents spatial interrelationship between pixels within a digital image.

Segmentation techniques include manual, semi-automatic, and fully automatic methods. Manual segmentation is time-consuming, which requires a trained observer to minimize the impact of inter-operator variability. In some cases, manual segmentation can be applied multiple times to ensure accuracy and reproducibility, particularly if the border of the lesion is unclear or if multiple focal lesions are observed at the same slice. Semi- or fully automatic segmentation is appealing, since each represents a more reproducible and faster way to enable observation of robust features from a given ROI. The Ischemic Stroke Lesion Segmentation (ISLES) challenge, a public competition that aims to explore the optimum segmentation method for stroke lesions, to some extent, promotes the development of tools for automation (www.isles-challenge.org/). Segmentation of the infarction core or peripheral penumbra on MR images can now be performed in a semi- or fully automated fashion in the context of machine learning [34-37]. For an ICH on CT images, automated segmentation algorithms based on random forest and open-source software (i.e., 3D-Slicer) have shown improved accuracy for determining hematoma volume over that of the conventional ABC/2 formula [38, 39]. Currently, the addition of manual curation to computer-aided edge detection is considered an ideal segmentation method with high reproducibility [16].

### 1.3 Feature extraction

Vast arrays of quantitative features can be extracted from multimodal images, which are broadly categorized as semantic metrics and agnostic metrics [16, 40]. Semantic metrics refer to a set of features that can be captured by the naked eye and are commonly used in the radiology lexicon to describe the lesion’s appearance characteristics. For example, “round”, “oval”, and “lobulated” are semantic descriptors of shape, which can help radiologists determine the irregularity of the lesion. Other semantic descriptors include size, location, and vascularity.

Agnostic metrics are calculated by mathematical algorithms and constitute the main body of radiomic features. Unlike semantic features, they cannot be obtained through visual interpretation. Agnostic metrics typically comprise first-, second-, and high-order statistical measures. First-order statistical measures describe the distribution of gray-level frequency from the pixel intensity histogram in a given ROI, without accounting for spatial interrelationships between pixels (e.g., maximum, median, skewness, and uniformity). Second-order statistical measures, that is, texture features, take into account both pixel intensity and statistical interrelationship in space (distance or orientation). They are the most widely used features in radiomics (Fig. 2) because texture analysis readily allows quantification of the heterogeneity within a lesion [32, 41, 42]. Texture features include co-occurrence matrix-based entropy, homogeneity, dissimilarity, correlation, etc. High-order statistical descriptors, such as busyness, contrast and coarseness, are calculated on matrices that consider relationships between 3 or more pixels.

In addition, radiomic analysis also employs a series of high-order statistical methods based on image filtration. Wavelet-transformed and Laplacian of Gaussian bandpass filters are the 2 most commonly used techniques [42, 43]. The former approach can convert spatial information into frequency and/or scale information in the direction of linear or radial waves (wavelet features) [44] , while the latter allows extraction of texture features with different coarseness levels from a prescribed ROI while denoising and enhancing the edge of the images [45]. Several radiomics studies on neuroimages have used these filtration techniques to distinguish stable hematomas from those prone to enlargement [46-49].

### 1.4 Exploratory analysis and modeling

By making full use of various extraction techniques, the quantity of radiomic features is endless, in theory. However, the increased risk of redundancy among features may lead to overfitting, reducing the generalization and robustness of the model. Hence, an exploratory analysis should be performed before modeling.

#### Feature selection

The core work in exploratory analysis is feature selection. For the internal feature-selection method, clustering analysis can be used as a first step. This method enables highly correlated radiomic features to be simplified into archetypal features per cluster, thus revealing the general distributive tendencies of the data. When multiple sets of data are available (two or more), the intraclass correlation coefficient (ICC) is usually used to detect stable features (ICC > 0.8) and exclude those that are susceptible to the variation of ROI segmentation [17]. In the external-selection process, radiomic data should be analyzed along with other pertinent clinical data in a prediction model with a well-defined endpoint. Radiomic features that are highly correlated with clinical features cannot provide additional information and should be excluded before modeling. According to the clinical endpoint, univariate filters such as Student's t test or Mann-Whitney test, can sort features by quality [50]. The least absolute shrinkage and selection operator (LASSO) is a method commonly used for dimensionality reduction and allows the selection of several informative features for modeling [47]. In radiomic analysis, it is necessary to combine the internal and external feature-selection methods to avoid redundancy and overfitting as much as possible.

#### Modeling

The ultimate goal of radiomics is to establish a practical and accurate model for predicting clinical outcomes (e.g., post-stroke death). In order to mine the full potential of existing datasets and explore the optimal model, clinical data should be added to the modeling process along with radiomic data. In addition, several machine-learning based modeling techniques can be employed.

**Table 1:** Scopes and Specifications of Radiomic Studies in Stroke.

Reference	Study Design	Image Modality	No. of Patients	No. of Features (Type)	Statistical Method	Clinical Utility
*Ischemic Stroke*						
Oliveira et al [56]	Retrospective Single-center	Non-contrast CT	10	4 (texture)	Univariate analysis	Diagnosis of acute stroke lesions
Peter et al [57]	Retrospective Single-center	Non-contrast CT	139	18 (texture)	Machine learning	Diagnosis of acute stroke lesions
Sikio et al [58]	Retrospective Single-center	T2W MRI	30	4 (texture)	Univariate analysis	Diagnosis of chronic stroke lesions
Ortiz-Ramon et al [59]	Prospective Single-center	MRI (FLAIR, T1W and T2W)	100	114 (texture and wavelet)	Machine learning	Diagnosis of chronic stroke lesions
Kassner et al [60]	Retrospective Single-center	Postcontrast T1W MRI	34	6 (intensity and texture)	Logistic regression	Prediction of hemorrhagic transformation
Qiu et al [61]	Retrospective Single-center	Non-contrast CT/CTA	67	326 (shape, size, intensity and texture)	Machine learning	Prediction of early recanalization
Tang et al [62]	Retrospective Multi-center	MRI (CBF and ADC)	155 (84+71)	456 (shape, intensity and texture)	LASSO algorithm	Evaluation of functional outcomes (7d/3M)
Cui et al [63]	Retrospective Single-center	MRI (DWI and PWI)	70 (40+30)	251 (intensity, texture and high-order)	Machine learning	Evaluation of functional outcomes (3M)
Betrouni et al [64]	Prospective Multi-center	T1W MRI	160	11 (intensity and texture)	Machine learning	Evaluation of cognitive impairment (6M)
*ICH*						
Zhang et al [66]	Retrospective Single-center	Non-contrast CT	261 (180+81)	576 (shape, intensity, texture, wavelet and high-order)	Machine learning	Diagnosis of AVM-related hematomas
Barras et al [70]	Prospective Multi-center	Non-contrast CT	81	5 (intensity)	Logistic regression	Prediction of early hematoma expansion
Shen et al [46]	Retrospective Single-center	Non-contrast CT	108 (81+27)	12 (texture and high-order)	Logistic regression	Prediction of early hematoma expansion
Ma et al [47]	Retrospective/ Multi-center	Non-contrast CT	254 (149+105)	576 (shape, intensity, texture, wavelet and high-order)	LASSO algorithm	Prediction of early hematoma expansion
Li et al [48]	Retrospective Single-center	Non-contrast CT	167	1227 (shape, size, intensity, texture, wavelet and high-order)	Machine learning	Prediction of early hematoma expansion
Xie et al [49]	Retrospective/ Single-center	Non-contrast CT	251 (177+74)	1942 (shape, size, intensity, texture and high-order)	LASSO algorithm	Prediction of early hematoma expansion
Yao et al [31]	Retrospective Single-center	Non-contrast CT	120 (80+40)	300 (Not Specified)	Machine learning	Prediction of early edema area
*SAH*						
Kanazawa et al [71]	Retrospective Single-center	Non-contrast CT	40	4 (intensity)	Logistic regression	Prediction of functional outcomes at discharge

Data in parentheses behind total number of patients indicates the quantities assigned to the training and validation cohorts in radiomics studies. ICH, intracerebral hemorrhage; SAH, subarachnoid hemorrhage; CTA, computed tomography angiography; CBF, cerebral blood flow; ADC, apparent diffusion coefficient; DWI, diffusion-weighted imaging; PWI, perfusion-weighted imaging. LASSO, least absolute shrinkage and selection operator.

Machine learning algorithms, such as support vector machines, artificial neural networks, and random forests, are supervised learning approaches that need to predefine a clinical label within a large number of training samples to train the model. Subsequently, the trained model is introduced to a testing set for performance evaluation. In contrast to supervised learning, unsupervised learning approaches (e.g., k-means clustering) can construct a model without clinical labels and using a limited sample size. Nevertheless, the performance of this model is limited by insufficiently labeled data. As a trade-off between high performance and low sample requirements, a growing number of semi-supervised learning models have recently been developed [18]. This method divides the modeling process into a supervised model training phase and an unsupervised feature learning phase, thus creating a balance between supervised and unsupervised learning. The choice of modeling techniques in radiomic analysis is important, in that different techniques may introduce discrepancies in model performance [51]. Therefore, multiple machine learning algorithms should be used for data mining, followed by the use of the optimal algorithm for modeling.

Validation is an indispensable step when constructing a prediction model for clinical applications. This could be carried out in either an internal or an external dataset. An externally validated model is considered more credible than an internally validated model, since independently obtained data can strengthen the validation [52]. Model performance is typically characterized by 2 attributes: discrimination and calibration [53]. Discrimination refers to how well the model distinguishes between those who are at high risk of a clinical event and those who are at low risk, and is often measured by means of receiver operating characteristic (ROC) curves, of which the area under the curve (AUC), sensitivity, and specificity are the 3 most commonly used indices. Calibration is a measure of agreement between the model’s predicted risk and observed risk, which can be reported in the manner of visuals (calibration curve) or statistical tests (e.g., the Hosmer-Lemeshow test). A reliable and applicable model should have both favorable discrimination and calibration and should exhibit statistical consistency between the training and validation datasets.

## 2. Applications of radiomics in stroke

With the advent of radiomics, additional information provided by quantitative image analysis has become an important supplement to traditional radiologic characteristics, accelerating the development of precision diagnosis and treatment in oncology [18]. Over the past few years, researchers have attempted to apply radiomics to stroke in the hope of gaining benefits similar to those obtained in cancer management. Radiomic data are expected to improve early decision-making for post-stroke patients. Currently, some progress has been made in both fields of ischemic and hemorrhagic strokes, and these applications seem to fall into 3 aspects: diagnosis of stroke lesion, prediction of early outcome, and long-term prognosis evaluation (Table 1).

### 2.1 Applications in ischemic stroke

To distinguish ischemic stroke from transient ischemic attack, an updated definition of ischemic stroke is an acute episode of neurologic deficit lasting longer than 24 h, or the presence of any imaging evidence (CT and/or MRI) of infarction directly related to the symptoms [1]. Ischemic stroke is the primary subtype of stroke that accounts for approximately 85% of all cases, and one-third of stroke patients will be permanently disabled [54].

#### Diagnosis of stroke lesions

Clinically, NCCT remains the first choice for patients with suspected stroke because it is efficient, non-invasive, and low in cost. Thrombolytic treatment with tissue plasminogen activator (tPA) in ischemic stroke patients benefits markedly from intravenous administration within 4.5h after symptom onset [55], which relies on early identification of stroke lesions by NCCT scans. In the acute phase, however, the changes in the ischemic area on NCCT images are often too subtle to be captured visually. In a study of 10 sex-and age-matched subjects (5 patients and 5 controls), Oliveira et al. [56] performed a quantitative texture analysis to distinguish healthy tissue from regions affected by AIS. They found that the gray-level co-occurrence matrix (GLCM)-based tissue texture parameters were significantly different between patients and controls, and the most discriminative feature was angular second moment. In another study of 139 patients with hyperacute ischemic stroke (< 8 h), the authors identified 6 texture features from NCCT images that could differentiate ischemic lesions from their contralateral normal tissue [57]. The classification model, constructed by 3 supervised machine learning algorithms (i.e., support vector machine, decision trees, and adaboost) achieved an average AUC of 0.82. In addition, they found that the size of the stroke lesion and classifier type did not affect the model performance.

Two other studies have investigated the diagnostic value of radiomics in stroke lesions using MR images. Sikio et al [58] evaluated 30 patients with chronic right hemisphere infarction and found that GLCM texture features derived from T2-weighted MR images were capable of revealing changes in both ischemic lesions and the ipsilateral structure outside the lesion (i.e., centrum semiovale). The ischemic region had lower homogeneity texture parameters than the unaffected side, but with relatively high values of complexity and randomness. Additionally, they found a close association between texture and diffusion tensor imaging (DTI) parameters (Pearson’s *r* > 0.5) in the ipsilateral mesencephalon and thereby concluded that texture analysis might be a useful tool for assisting the DTI method in the detection of chronic ischemic lesions. Ortiz-Ramon and colleagues [59] used multimodal MRI data of different brain tissues (i.e., white matter and subcortical structures) from 100 elderly individuals to explore whether radiomic analysis could distinguish patients who had a prior ischemic stroke from a healthy population that was stroke-free. They found that radiomic features, including texture and wavelet, could robustly identify the presence of previous stroke lesions with favorable discrimination (AUC > 0.7), irrespective of MRI sequence used, tissue type, and stroke subtype.

#### Prediction of early outcomes

The extra benefit of intravenous tPA for AIS comes from exclusion of patients with a high risk of secondary intracranial hemorrhage, which is a serious complication, primarily due to damage of the blood-brain barrier. Kassner et al [60] conducted a comparative study to assess the predictive ability for early hemorrhagic transformation after AIS (< 72 h) between texture parameters and visual enhancement score in postcontrast T1-weighted MR images. Texture features (contrast and correlation) were found to be more predictive of hemorrhagic transformation than visual evidence of enhancement (AUC < 0.6), with an AUC > 0.75. This result is promising, because texture analysis may help select eligible patients who are most likely to benefit from thrombolytic treatment. For thrombus in the internal carotid artery and M1 middle cerebral artery segment, recanalization with thrombolytics within these proximal intracranial arterial segments is rare. Qiu et al [61] performed a radiomic analysis to predict early recanalization after proximal occlusion in large vessels in 67 AIS patients administered intravenous alteplase. They concluded that the combination of radiomic features from NCCT, CTA, and radiomic changes (i.e., CTA-NCCT) was more predictive of early recanalization (AUC = 0.85), compared with solely conventional thrombus imaging characteristics, such as length, volume, or permeability.

#### Evaluation of long-term prognosis

Accurate identification of a salvageable penumbra promises to improve decision-making during post-stroke management and extend the therapeutic time window. A multicenter study (n = 155) conducted by Tang et al [62] applied radiomic analysis to quantify the penumbra and core area from both the apparent diffusion coefficient and cerebral blood flow maps in patients with AIS (< 9 h). In the external dataset, the constructed radiomics nomogram could strongly predict favorable clinical outcomes at 7 days and at 3 months, with AUCs of 0.88 and 0.77, respectively. Thus, researchers concluded that radiomics has the potential to select patients for thrombolysis beyond the current time window. Another study based on multimodal MR images of AIS patients confirmed the prognostic value of radiomic features in predicting 3-month outcomes using a feature selection strategy combining redundancy reduction and informative degree evaluation [63].

Betrouni et al [64] found that texture features of MR images (i.e., kurtosis and inverse difference moment) in the hippocampus and entorhinal cortex at 72 h after stroke onset were significantly different between patients with and without 6-month cognitive impairment (CI). The prediction model built using a support vector machine showed excellent discrimination ability for CI (AUC > 0.9). This result was further confirmed in a rat model of middle cerebral artery transient occlusion, in which there was a significant correlation between texture features and neural density in the hippocampus contralateral to the ischemic region. These associations are based on the hypothesis that the mild neuron loss involved in post-stroke CI could be captured at an early stage by measuring changes in image gray values. The results of this study are relatively reliable because of synthesized clinical and preclinical evidence and indicate that the MR texture features may be an early imaging marker for screening of long-term CI in patients with AIS.

### 2.2 Applications in hemorrhagic stroke

Hemorrhagic stroke subtypes include ICH and SAH, both of which are not caused by trauma. Although hemorrhagic stroke accounts for only a small proportion of stroke cases (ca. 15%), its associated disability and mortality are high. It is reported that ICH alone has a fatality rate of 40% at 1 month [65].

#### Diagnosis of stroke lesions

In clinical scenarios, the diagnosis of ICH caused by arteriovenous malformation (AVM) relies to a large extent on the CTA technique, since AVM-related hematomas are difficult to distinguish on NCCT images from those triggered by hypertension or cerebral amyloid angiopathy. However, angiography entails a large investment of time and resources, and may not be widely available or routinely performed in primary medical institutions. Early and accurate diagnosis of AVM-related hematomas is crucial for guiding treatment decisions, for instance, deciding whether or not to embolize the nidus to avoid rebleeding. Zhang and colleagues [66] performed a radiomics study to differentiate between acute ICH (< 6 h) of diverse etiologies on NCCT. The hypothesis driving this research is that AVM-related hematomas embedded in malformed vasculature are more heterogeneous in composition and could be identified through quantitative radiomic analysis. After integrating optimal feature selection and modeling algorithms, the established NCCT radiomics model could accurately diagnose AVM-ICH in the validation cohort (n = 81), with an AUC of 0.95, sensitivity of 88%, and specificity of 93%. In addition, radiomic analysis showed superior diagnostic performance to subjective assessment by interventional radiologists with different levels of work experience.

#### Prediction of early outcomes

Hematoma expansion (HE) following ICH reflects an active bleeding process, which is strongly associated with early neurological deterioration and poor long-term prognosis and is an appealing treatment target in clinical trials [67, 68]. In addition to the currently available clinical and radiologic risk factors [69], a growing body of evidence in radiomics has shown promise for predicting HE [46-49, 70].

In 2013, Barras et al [70] conducted the first radiomics study on NCCT images of acute ICH patients (< 3 h) and identified a histogram intensity feature (i.e., coefficient of variation) most relevant to HE. In 2018, Shen et al [46] used the Laplacian of Gaussian bandpass filter to extract a series of coarse to fine texture features from images to predict early HE, with AUC reaching 0.92. Recently, the favorable performance of the radiomics model for HE was confirmed in a multicenter study (n = 254) [47], wherein ICH data from 4 independent medical centers were analyzed. In another comparative study evaluating 251 patients with acute ICH, the radiomics model was found to be superior to the radiological model that incorporates conventional NCCT markers (AUC, 0.9 *versus* 0.8) [49]. Notably, the interpretation of results among these studies is limited by different HE definitions (> 6 mL, > 12.5 mL, or > 33%) and the non-standardized follow-up NCCT timing for HE detection. Besides the predictive value of ICH growth, radiomic signature has also been reported to be highly predictive of the edema area around the basal ganglia hematoma at an early stage [31].

#### Evaluation of long-term prognosis

Clinical data regarding the evaluation of prognosis in hemorrhagic stroke using radiomic analysis are scarce. However, recently, a pilot study conducted by Kanazawa et al [71] explored the feasibility of quantitative texture analysis of NCCT images in predicting clinical outcomes in 40 patients with aneurysmal SAH. They found that the mean CT value of subarachnoid bleeding at the level of the basal cisterna was the only texture feature independently associated with delayed cerebral ischemia and prognosis at discharge. At the optimal cutoff value of 53 HU, this parameter could predict poor functional outcomes (mRS ≥ 3) with a high specificity of 91.7%. Due to their qualitative nature, radiological markers such as the Fisher, Hijdra, and SEBES grading systems for measuring SAH severity, are susceptible to interobserver variability [72, 73]. This work is an initial step toward the development of a quantitative radiomics tool for the assessment of SAH prognosis on NCCT images. For ischemic and hemorrhagic stroke applications, the difference between CT- and MRI-radiomics is presented in Table 2.

**Table 2:** Comparison of CT- and MRI-radiomics in Stroke Application.

Neuroimaging	Technique	Study Population	Research Objective	Pros and Cons
CT	Non-contrast CT CTA	Acute ischemic stroke Intracerebral hemorrhage Subarachnoid hemorrhage	Diagnosis of acute stroke lesions Prediction of early outcomes	Convenient for image data acquisition and transformation Difficulty in ROI segmentation of acute ischemic stroke Limited source of radiomics information from lesions More interpretable for radiomic features
MRI	T1W/T2W/FLAIR Postcontrast T1W DWI (ADC) PWI (CBF)	Acute ischemic stroke Chronic ischemic stroke	Diagnosis of chronic stroke lesions Evaluation of long-term prognosis	Inconvenience in archiving and communication caused by relatively large image data Accurate 3D-ROI delineation of ischemic lesions Entail resampling intensity values for multimodal images Lack of interpretability of radiomic features

CTA, computed tomography angiography; DWI, diffusion-weighted imaging; ADC, apparent diffusion coefficient; PWI, perfusion-weighted imaging; CBF, cerebral blood flow; ROI, region of interest.

## 3. Challenges and Future Directions

Several challenges currently exist around radiomics, facing its widespread use in stroke management. One of the most critical challenges is the lack of reproducibility among extracted radiomic features, which arises primarily from the variability in radiomic workflow. Since radiomics studies can be performed either on open-source software platforms (e.g., MaZda or IBEX) or with in-house developed tools in the MATLAB environment, the pre- and post-processing techniques, the segmentation method for the ROI, and the type and quantity of extracted radiomic features are significantly different among these investigations. This contributes to difficulties in reproduction and comparisons between studies, as well as in the integration of results for meta-analysis. To normalize the reporting process and improve repeatability, investigators have proposed the radiomics quality score (RQS) to help ascertain whether a radiomics study conforms to best-practice procedures [20]. More recently, the image biomarker standardization initiative (IBSI) has also been put forward, offering a standardized general image processing workflow for quantitative radiomic analysis [74]. Future radiomics studies need to be carried out in the light of these reporting guidelines.

As mentioned earlier, imaging protocols highly affect the stability of radiomic features and, thus, may be another factor that introduces non-reproducibility in radiomics. It has recently been reported that CT image acquisition and reconstruction parameters could cause variation in the values of a considerable number of features [22, 25, 26]. This is understandable, considering that a greater slice thickness may increase the error in ROI delineation due to the partial volume effect. Although some efforts have been made to improve the robustness of radiomic features, such as the use of preprocessing techniques of filtration or gray-level normalization, achievements are extremely limited, particularly in stroke. The Quantitative Imaging Network (QIN) is a cooperative project initiated by the National Institutes of Health (NIH) in an effort to develop new quantitative imaging tools and methods for cancer management in clinical trials; one of its goals is to promote the standardization of quantitative imaging protocols in cancer [75]. For radiomics studies of stroke, there is a pressing need to develop standardized MRI and CT scanning protocols that are widely acceptable in the research community.

While NCCT-based radiomic features have the ability to identify acute infarcted tissue, the information they capture is confined to a part of the lesion in a single slice (2D-radiomics) [56, 57]. It is challenging to perform a volumetric assessment for stroke lesions that cannot be visually perceived. Although MR diffusion and perfusion imaging perform well in this regard, NCCT is increasingly favored in routine practice because of its speed, low cost, and lack of contraindications. Therefore, future research should focus on developing semi- or full-automatic segmentation tools for acute stroke lesions on NCCT images. Interestingly, 3D-volumetric measurements in ICH and SAH have achieved automatic segmentation with the aid of machine learning [39, 76]. The next step is to determine the best way to integrate radiomic data from multiple hemorrhage lesions. For example, it remains unclear whether radiomic features derived from blood clots in different cerebral cisterns or at multiple locations (e.g., brain stem and cerebellum) should be summed, averaged, or weighted. This issue exists widely in the radiomic analysis of other lesion types, and thus, the solution is of great significance.

Another major challenge in radiomic analysis is the need for large-scale data. Statistically adequate radiomic data are conducive to generating a classifier model with high robustness and generalization. However, the creation of databases is time-consuming because of the collection of medical images and 3D-ROIs segmentation. Although deep learning algorithms based on convolutional neural networks can automatically extract features from an unsegmented image, the requirements for expensive hardware and immense volumes of annotated data have limited their applicability [77-79]. Consequently, data sharing among different research organizations and medical centers is crucial in radiomics [13, 16]. Currently, most of the available radiomic evidence regarding stroke is derived from retrospective and single-center studies, which leads to an unstable association of radiomic features with clinical events, due to selection bias. For instance, the type of radiomic features that could predict early HE of acute ICH differed markedly across studies [47-49]. Shared databases consisting of radiomic data and other pertinent medical information (e.g., clinical or demographic data) from multiple centers, after removing the data management hurdle, may serve as an external tool for validating the credibility of existing results and eventually promote the standardization of radiomics research in stroke around the world.

## 4. Conclusions

With advances in computational technologies, radiomic analyses of stroke neuroimages have been applied successfully to NCCT and MRI scans. The derived radiomic signature could potentially be used to diagnose stroke lesions, predict early transformation, and evaluate long-term prognosis after stroke onset. Despite these promising results, various challenges need to be addressed before its widespread use as a clinical tool. These challenges seem to arise from the reproducibility of study results, standardization of protocols in radiomic workflow, and data sharing among different medical institutions. By mining the full potential of radiomics in the field of stroke, it is expected to optimize secondary prevention strategies and facilitate the development of personalized precision medicine in post-stroke patients.
